# Immunogenicity and Acceptance of Influenza A (H1N1) Vaccine in a Cohort of Chronic Hepatitis C Patients Receiving Pegylated-Interferon Treatment

**DOI:** 10.1371/journal.pone.0048610

**Published:** 2012-11-08

**Authors:** Manuel Hernández-Guerra, Yanira González-Méndez, Patricia de Molina, Antonio Z. Gimeno-García, Marta Carrillo, Carlos Casanova, Tomás Pumarola, Alejandro Jimenez, Miriam Hernández-Porto, Álvaro Torres, Enrique Quintero

**Affiliations:** 1 Liver Unit, University Hospital of the Canary Islands, Tenerife, Spain; 2 Research Unit, University Hospital of the Canary Islands, Tenerife, Spain; 3 Barcelona Centre for International Health Research (CRESIB), Hospital Clínic-Universitat de Barcelona, Barcelona, Spain; 4 Department of Clinical Microbiology, Hospital Clínic, School of Medicine, University of Barcelona, Barcelona, Spain; 5 Preventive Medicine and Public Health Department, University of La Laguna, Tenerife, Spain; University of Sydney, Australia

## Abstract

**Background & Aims:**

Individuals at risk of (H1N1) influenza A infection are recommended to receive vaccination. Chronic hepatitis C (CHC) patients receiving treatment might be at a higher risk of respiratory bacterial infections after influenza infection. However, there are no observational studies evaluating the immunogenicity, tolerance and acceptance of 2009 influenza A vaccine in CHC patients.

**Methods:**

We evaluated the immunogenicity of influenza A vaccine (Pandemrix®) by using the hemagglutination inhibition (HI) titers method in a well defined cohort of CHC patients receiving or not receiving pegylated-interferon and ribavirin, and compared it with healthy subjects (controls). A group of patients with inflammatory bowel disease (IBD) under immunosuppression, thought to have a lower immune response to seasonal influenza vaccine, were also included as a negative control group. In addition, tolerance to injection site reactions and acceptance was assessed by a validated questionnaire (Vaccinees' perception of injection-VAPI-questionnaire).

**Results:**

Of 114 subjects invited to participate, 68% accepted and, after exclusions, 72 were included. Post-vaccination geometric mean titers and seroprotection/seroconversion rates were optimal in CHC patients with ongoing treatment (n = 15; 232, CI95% 46–1166; 93%; 93%), without treatment (n = 10; 226, CI95% 69–743: 100%; 100%) and controls (n = 15;168, CI95% 42–680; 93%; 86%) with no differences between groups (*P* = 0.8). In contrast, IBD patients had a significantly lower immunogenic response (n = 27; 60, CI95% 42–680;66%;66%; *P* = 0.006). All the groups showed a satisfactory tolerance although CHC patients with ongoing treatment showed more local discomfort after vaccine injection.

**Conclusion:**

There appeared to be no differences between CHC patients and healthy controls in serological response and acceptance of (H1N1) influenza vaccination.

## Background

Physicians who care for patients with chronic digestive disease were recommended by the World Health Organization to encourage patients to receive the novel (H1N1) influenza A vaccine during the global pandemic of 2009. The recommendations concerned elderly patients (>65 years) and those with chronic medical conditions or immunosuppression [1], considered to be at high risk of developing influenza-related complications [2]. The latter conditions are important in chronic hepatitis C (CHC) patients, especially those receiving standard medical treatment (pegylated-interferon and ribavirin). Indeed, hepatologists are aware that CHC patients may experience bacterial infections during pegylated-interferon based regimens related or not to neutropenia[3]–[6]. During the 2009 (H1N1) influenza A virus outbreak, scarce data were available to reassure CHC patients regarding tolerance and serological response to the vaccine. This provoked anxiety in patients potentially at risk of severe infection and even among physicians without guidelines to follow.

In addition, CHC patients with ongoing pegylated-interferon based therapy may have a lower immunogenic response [7] and experience side effects that may be aggravated by vaccination adverse effects, thus compromising CHC treatment adherence.

Therefore, the present study was conducted to evaluate the (H1N1) influenza A virus vaccine immunogenic response in CHC patients with and without ongoing standard medical treatment and compared it with that of healthy subjects.

Recently, a lower immunogenic response has been found in pediatric patients with inflammatory bowel disease (IBD) under immunosuppression therapy [8]. Therefore, an additional group of patients with IBD were included. In addition, perception and acceptance of influenza vaccination was assessed using a validated outcome questionnaire designed for this purpose [9].

## Methods

### Ethics Statement

Patients and a group of healthy volunteer healthcare workers were invited to participate and enrolled after written informed consent was obtained. Approval for the study protocol was obtained from the national Agencia Española del Medicamento y Productos Sanitarios and local ethics committee (Hospital Universitario de Canarias), and the study was conducted in accordance with the principles of the 1975 Declaration of Helsinki.

### Patients and methods

As standard of care, vaccination against (H1N1) influenza A was offered to adult (≥18 years of age) patients with CHC, referred for hepatitis C virus treatment assessment, and IBD patients receiving immunosuppression therapy during at least 3 months. They were recruited consecutively during outpatient visits at the University Hospital of the Canary Islands between November 2009 and March 2010, and followed during at least 6 months.

We excluded patients who had previously been vaccinated against 2009 (H1N1) influenza A, those with documented (H1N1) influenza A infection, a known allergy to eggs or other components of the vaccine, or pregnancy. Previous seasonal influenza vaccination was not an exclusion criterion. Reasons against vaccination given by patients who refused to participate were also recorded.

Medical records were used to retrieve information on hepatitis C virus regarding virus genotype, viral load and other hematological parameters. In those patients receiving hepatitis C virus treatment, the type of pegylated-interferon, ribavirin dose and sustained virological response (SVR) were recorded. Concerning IBD patients, we also recorded the type of disease and immunosuppression treatment at the time of vaccination (azathioprine/6-mercaptopurine, methotrexate or anti-tumour necrosis factor agents) as well as blood test results.

### Immunogenicity assessment

In patients and healthy volunteer healthcare workers, vaccination was administered by intramuscular injection in the deltoid region of the non-dominant arm with a single (0.5 ml) dose of adjuvanted influenza A (A/California/7/2009 H1N1-vlike strain) 2009 vaccine (Pandemrix®, GlaxoSmithKline, Brentford, United Kingdom). Two blood samples per participant were drawn: one before vaccination and one at least 3 weeks after vaccination (3–13 weeks). Serum was stored at −80°C until measurement of hemagglutination inhibition (HI) titers.

Samples were sent on dry ice to the Department of Clinical Microbiology, Hospital Clínic, Barcelona. All samples were coded and the laboratory was blinded to the identity and clinical details of the subjects.

Influenza-specific antibody levels were measured using HI assay with chicken red blood cells according to the World Health Organization standardized protocol [10]. In brief, serum non-specific inhibitors were treated with receptor destroying enzyme overnight at 37°C, followed by inactivation at 56°C for 30 min. The standard antigen was diluted to contain four hemagglutinin units and back titration was performed. Two-fold serial dilution of RDE-treated sera was performed in v-bottom microtiter plates. Then, diluted sera were mixed with 25 μl of H1N1pdm antigen (2010–2011 World Health Organization influenza reagent kit for identification of influenza isolates). After 1 hour incubation at room temperature, 50 μl of red blood cell (diluted 0.05% in PBS) was added to the wells. Positive and negative serum controls were included for each plate. Titers were expressed as the reciprocal of the highest dilution of serum that inhibited hemagglutination.

HI antibody titers were summarized with the criteria conventionally used to assess the immunogenicity of influenza vaccines: geometric mean titer (GMT), geometric mean titer ratio (GMTR), seroprotection rate (proportion with titers ≥1∶40), seroconversion rate (proportion with prevaccination titers <1∶10 and a postvaccination titer ≥1∶40, or a prevaccination titer ≥1∶10, and ≥4-fold increase after vaccination) [11].

### Acceptance and tolerance

To assess how injection site reactions are perceived and how this perception affects acceptance of vaccination and willingness to be vaccinated in the future, a structured, self-administered questionnaire designed for this purpose was given to patients and completed 21 days after vaccination. The vaccinees' perception of injection questionnaire (VAPI questionnaire; with permission of Sanofi Pasteur) [9] was developed to assess subjects' perception and attitudes concerning influenza vaccination and any injection site reactions that may occur. In brief, the VAPI questionnaire comprises 4 dimensions (“bother from injection site reaction”; “arm movement”; “sleep”; “acceptability”) and a number of items each measuring a different aspect of subjects' perceptions following injection. Each question is answered by selecting a response from a five-point rating scale (1, Not at all; 2, A little; 3, Moderately; 4, Very; 5, Extremely) and yes or no when appropriate.

In addition, systemic adverse events commonly associated with influenza vaccine were recorded (fever, malaise, nausea/vomiting, diarrhea, headache, myalgia/arthralgia, irritability and somnolence) occurring within 21 days and serious adverse events or death within 6 months of vaccination.

### Statistical analysis

The baseline and post-vaccination GMT and GMTR of HI antibody titers were obtained for each group. After verifying normal distribution of the data with Kolgomorov-Smirnoff test, Log HA antibody titers were compared using ANOVA, and post-hoc comparisons were carried out with Tukeýs HSD test. HI antibody titers below 1∶10 were assigned a value of 1∶5 for the purposes of calculations.

Qualitative data are expressed as frequencies and percentages. The proportions of seroprotection, seroconversion and SVR rates were compared between groups by Jonckheere-Terpstra test. After verifying normal distribution of the data with Kolgomorov-Smirnoff test, the mean scores of VAPI questionnaire dimensions were compared using ANOVA and post-hoc comparisons were carried out with Tukeýs HSD test or Kruskall-Wallis test when appropriate.

Statistical analysis was performed using the SPSS 15.0 for Windows statistical package (SPSS Inc., Chicago, IL) and StatXact-5.0.3 (Cytel CO, MA). Differences with a *p* value less than 0.05 were considered significant.

## Results

### Characteristics of the study groups

One hundred and fourteen patients (aged 41.3±11.4 years, 48% female) were asked to participate in the study. Thirty seven patients (32%) refused to participate; the most common reasons for refusing the (H1N1) influenza A vaccine are shown in table 1. No statistical differences were found between groups (*P* = 0.20). Sixteen patients were excluded because of previous (H1N1) influenza A vaccination, one patient was pregnant and three had documented (H1N1) influenza A infection. Finally, 72 patients consented to participate and received vaccination (Table 2). All the patients were of European descent. At the time of inclusion, 30% of the participating patients had received seasonal influenza vaccination.

**Table 1:** Reasons given for declining 2009 (H1N1) influenza A vaccination.

	CHC with ongoing treatment, n (%)	CHC without treatment, n (%)	IBD patients, n (%)
**Worry about side effects**	1 (100)	2(33.3)	8 (26.7)
**Never receives seasonal influenza vaccine**	0	2(33.3)	6 (20)
**Query on the efficacy of the vaccine**	0	2(33.3)	3 (10)
**Simply did not want the vaccine**	0	0	13 (43.3)

CHC, chronic hepatitis C; IBD, inflammatory bowel disease.

**Table 2:** Demographic data and baseline characteristics of the participants according to group.

	CHC with ongoing treatment (n = 15)	CHC without treatment (n = 10)	IBD patients (n = 32)	Controls (n = 15)
**Gender**, ***female*** **(%)**	4 (27)	3 (30)	17 (53)	11(73)
**Age, years**	47.4±9.5	42.4±10.9	36.3±9.6	38.8±10.5
**BMI (Kg/cm^2^)**	23.4±3.5	23.9±3.2	24.9±5.0	22.0±2.9
**Viral load (IU)**	44408±154991	1486684±1866724	-	-
**Genotype 1 and 4, n (%)**	11 (73)	9 (90)	-	-
**Type of IBD, Crohńs disease n (%)**	-	-	27 (84)	-
**Type of immunosuppression treatment in IBD patients, n (%)**				
** Azathioprine/6-mercaptopurine**	-	-	29 (91)	-
** Methotrexate**	-	-	3 (9)	-
** Anti-tumour necrosis factor agents**	-	-	15 (47)	-
**Hemogram**				
** Leucocytes (10^9^/L)**	4.0±2.2	7.1±1.7	6.2±1.6	-
** Neutrophils (10^9^/L)**	2.3±1.4	3.4±1.4	3.9±1.2	-
** Lymphocytes (10^9^/L)**	1.1±0.6	2.9±0.9	1.7±0.9	-
** Hematocrit (%)**	39.3±4.0	44.0±3.0	40.5±4.4	-
** Platelets (10^9^/L)**	159±54	207±61	264±117	-
**Liver function tests**				
** AST (IU)**	30±14	112±112	23±11	-
** ALT (IU)**	29±21	201±256	20±16	-

CHC, chronic hepatitis C; IBD, inflammatory bowel disease; BMI, Body mass index; AST, aspartate aminotransferase; ALT, alanine aminotransferase.

Mean ± standard deviation.

Fifteen healthy individuals also took part in the study and blood samples were collected.

Regarding the grade of fibrosis in CHC patients, although liver biopsies were only available in four CHC patients with ongoing treatment during vaccination (METAVIR score A1F2, A1F1, A2F3 and A1F1) and two CHC patients not receiving treatment (METAVIR score A2F4, in both), the rest of the patients did not have biochemical (low albumin or prothrombin time, and high bilirubin) or ultrasonographic (liver surface nodularity, parenchymal nodularity, or atrophy of the right lobe) signs of cirrhosis. In addition, noninvasive tests to predict liver fibrosis such as Forns index of fibrosis [12], AST to platelet ratio index (APRI) [13]and FIB-4 [14] all showed moderate scores in both groups.

### Post-vaccination geometric mean titers and seroprotection/seroconversion rates

Blood samples were available in 67 subjects (5 patients did not have baseline serum).

The global median time between baseline and post-vaccination serum sampling during follow-up was 6 weeks (range 3–13). There were no differences between a) controls [4 (range 4–11)] and CHC group [5 (4–11)]; *P* = 0.65 and b) controls and IBD group [7 (range 3–13)]; *P* = 0.28. In each group, only one subject had blood analysis out of the 3–9 week interval.

At baseline, antibodies against the vaccine strain were detected (titer ≥1∶10, but only one higher than 1∶40) in 11 subjects (CHC patients with ongoing treatment, n = 3; CHC patients without treatment, n = 2; IBD group, n = 4 and controls, n = 2).

The overall post-vaccination GMT was 124 (95% CI 25–619), representing a 17.9-fold increase from the pre-vaccination level. The post-vaccination GMT was higher in the group of CHC patients than in the IBD patients (229, 95% CI 55 to 957 vs. 60, 95% CI 12 to 307; *P* = 0.006). However, there were no differences between CHC patients with ongoing treatment compared with CHC patients without treatment or controls (Table 3, *P* = 0.89). Results expressed as GMTR showed similar results (Table 3). The post-vaccination GMT for IBD patients with mono-immunotherapy (n = 14) and combined immunosuppression (n = 13) was 44 (95% CI 12 to 157) and 84 (95% CI 12 to 595)(*P* = 0.32), representing a 7.4 and 9.6-fold increase, respectively.

**Table 3:** Antibody responses after vaccination according to group.

	CHC with ongoing treatment (n = 15)	CHC without treatment (n = 10)	IBD patients (n = 27)	Controls (n = 15)
**GMT post-vaccination**	232 (46–116)	226 (69–743)†	60 (12–307)*	168 (42–680)
**GMTR** &	43 (10–180)	32 (7–137)†	15 (4–64)^‡^	24 (7–78)
**Seroprotection**	14/15, (93.3)	10/10, (100)	18/27, (66.7)^¶^	14/15, (93.3)
**Seroconversion**	14/15, (93.3)	10/10, (100)	15/26, (66.7)**	6/7, (85.7)

CHC, chronic hepatitis C; IBD, inflammatory bowel disease; GMT, Geometric mean titer (IC 95%); GMTR, Geometric mean titer ratio (IC 95%); Seroprotection, n (%); Seroconversion, n (%).

& One IBD patient and 4 control subjects did not have pre-vaccination serum sample for GMTR calculation.

† *P* = 0.8 vs. CHC with ongoing treatment; *P = 0.006 vs. CHC patients; ^‡^ *P* = 0.005; ^¶^ *P* = 0.02 vs. CHC patients and controls; **P = 0.01 vs. CHC patients and controls.

The overall proportion with seroprotection and seroconversion was not different between CHC groups and controls (Table 3). However, IBD patients showed a significantly lower percentage of post-vaccination seroprotection (*P* = 0.02) and seroconversion rate (*P* = 0.01).

IBD patients on a single immunosuppressive agent had a similar response rate to those on combined immunosuppression (seroprotection: 10/14, 71.4% vs. 8/13, 61.5%, *P* = 0.45 respectively; seroconversion: 9/14, 64.3% vs. 6/12, 50%, respectively; *P* = 0.37).

### Acceptance, tolerability and adverse events of vaccination

The majority of consenting patients completed the VAPI questionnaire (83%, Table 4). CHC patients with ongoing therapy scored the highest with respect to injection site reactions (inconvenience related to pain, redness, swelling, itching, hardening, bruising; and arm movement), with significant differences compared with untreated CHC patients and IBD patients (Table 4). Regarding acceptability (use of analgesics or interference with concomitant treatment) and the remaining questions, a generally favorable response was observed. Most importantly, a low proportion of patients in all groups were actively against being vaccinated again next season.

**Table 4:** Comparison of VAPI scores between groups of patients.

	CHC with ongoing treatment (n = 14)	CHC without treatment (n = 9)	IBD patients (n = 24)	*P* value
**Bother** a	2.9±1.4†	1.7±0.8	2.0±1.0	0.02
**Arm movement**	3.3±1.3* ^‡^	1.9±1.1	2.0±1.0	<0.01
**Sleep**	2.3±1.5	1.4±1.0	1.7±1.3	0.16
**Needed analgesics yes, n (%)**	6 (43)	1 (11)	4 (18)	0.14
**Stopped concomitant treatment yes, n (%)**	0 (0)	0 (0)	1 (4)	0.58
**Anxiety before vaccination yes, n (%)**	3(21)	3 (30)	4 (18)	0.75
**Discomfort during injection yes, n (%)**	3 (21)	2 (20)	5 (23)	0.98
**Willingness to be re-vaccinated (yes/no/unknown), n (%)**	5 (36)/3 (21)/6 (43)	8 (89)/0 (0)/1 (11)	14 (64)/4 (18)/4 (18)	0.11

CHC, chronic hepatitis C; IBD, inflammatory bowel disease.

One patient in each CHC group and 8 IBD patients did not complete the questionnaire.

a How the patient was bothered by pain, redness, swelling, itching, hardening, bruising at the vaccination site.

† *P*  = 0.04 vs. CHC patients without treatment; *P = 0.01 vs. CHC patients without treatment; ^‡^ *P* <0.01 vs. IBD patients.

Mean ± standard deviation.

Other systemic adverse events specifically assessed (fever, malaise, nausea/vomiting, diarrhea, headache, myalgia/arthralgia, irritability and somnolence) were not different between the groups (Table 5).

**Table 5:** Systemic adverse events within 21 days after vaccination in group of patients.

	CHC with ongoing treatment (n = 14)	CHC without treatment (n = 9)	IBD patients (n = 24)	*P* value
**Fever yes, n (%)**	1 (7)	0 (0)	0 (0)	0.30
**Malaise yes, n (%)**	2 (14)	1 (11)	2 (8)	0.23
**Nausea/Vomiting yes, n (%)**	0 (0)	0 (0)	1 (4)	0.61
**Diarrhea yes, n (%)**	1 (7)	0 (0)	2 (8)	0.67
**Headache yes, n (%)**	1 (7)	0 (0)	1 (4)	0.68
**Myalgia/Arthralgia yes, n (%)**	2 (14)	0 (0)	3 (12)	0.50
**Irritability yes, n (%)**	1 (7)	0 (0)	3 (12)	0.50
**Somnolence yes, n (%)**	3 (21)	0 (0)	1 (4)	0.11

CHC, chronic hepatitis C; IBD, inflammatory bowel disease.

One patient in each CHC group and 8 IBD patients did not complete the questionnaire.

No deaths or serious vaccine-related adverse events were reported during follow-up.

Only one CHC patient with ongoing treatment (with post-vaccination seroprotection) reported symptoms of respiratory disease, although influenza A infection was not confirmed by laboratory tests.

### Effects of vaccination on virological response

Regarding the impact of influenza vaccination on SVR, no significant differences were found between CHC patients receiving standard medical care during vaccination (n = 15) compared to those treated after vaccination (n = 8). In addition, viral load, aspartate aminotransferase (AST) and alanine aminotransferase (ALT) values were not different 6 months after the end of treatment (Table 6). The groups were similar regarding prognostic factors of favorable outcome.

**Table 6:** Characteristics and type of response in group of patients with CHC after hepatitis C virus treatment.

	CHC with ongoing treatment (n = 15)	CHC treated after vaccination (n = 8)	*P* value
**Peg-interferon α-2a, n (%)**	12 (80)	4 (50)	0.13
**Dose of Peg-interferon (µcg)**	113±41	144±38	0.09
**Dose of ribavirin (mg)**	960±155	1000±185	0.58
**SVR, n (%)**	7/15 (46.7)	5/8 (62.5)	0.67
**Viral load (IU)**	578797±*1255219*	128152±*282710*	0.59
**AST (IU)**	33±*21*	34±*19*	0.63
**ALT (IU)**	35±*34*	36±*28*	0.72
**Forns fibrosis index**y	5.36±1.5	5.41±1.8	0.89
**APRI**	0.75±*0.37*	*0.95±0.40*	0.16
**FIB-4**	1.82±*0.64*	*2.96±2.17*	0.26

CHC, chronic hepatitis C; SVR, sustained virological response; AST, aspartate aminotransferase; ALT, alanine aminotransferase; APRI, AST to platelet ratio index.

Mean ± standard deviation.

## Discussion

Influenza virus infection can cause severe illness and mortality in high risk patients. Annual immunization is highly recommended in elderly subjects and adults with chronic medical conditions or immunosuppression, in order to decrease attributable morbidity and mortality. These recommendations were extended to the pandemic 2009 novel (H1N1) influenza A virus [1], [2]. Despite these firm recommendations by health authorities, a low rate of vaccination was expected. Indeed public anxiety about the safety of the novel vaccine reported in the media contributed. In fact, one-third of our patients refused to be vaccinated. The main arguments against were doubts about vaccine safety and side effects, and concern over vaccine efficacy. This is in keeping with other studies specifically addressing (H1N1) influenza A vaccine acceptance among patients and healthworkers [15]–[17]. Therefore, vaccine tolerance and efficacy studies focusing on specific groups of patients are of value in the event of a new influenza pandemic outbreak, especially since some relevant clinical trials evaluating the vaccine have excluded CHC patients [18], [19].

The infection rate among non-cirrhotic CHC patient receiving current antiviral treatment is 5–30%. This high incidence of infections has been associated to neutrophil impairment due to pegylated-interferon [20] more than to decreased neutrophil count [3]–[6], [21], [22]. Given that 20–40% of infections are of the upper respiratory tract, influenza vaccination should be recommended in these high-risk patients.

Regarding CHC patients and influenza vaccination, limited information is available and mostly related to advanced cirrhotic or liver transplant patients [23], [24]. Moreover, little is known about the immunogenic response of non-cirrhotic CHC patients. Theoretically, interferon alpha is a strong stimulator of immune response and for that reason it has recently been used as an adjuvant in influenza vaccines [25], [26]. On the other hand, cytotoxic T lymphocyte function is impaired by hepatitis C virus [27] and CHC patients may have a different T cells immune response to influenza A HA protein and other antigens used in vaccines, during interferon therapy for hepatitis C virus [7]. In addition, severe influenza infection has occurred after vaccination and doubts about vaccine effectiveness have been reported [28].

To our knowledge this is the first study to evaluate the immunogenicity, and perceived tolerance of the pandemic 2009 (H1N1) influenza A vaccine in a well defined cohort of CHC patients. Our findings are useful from the opportunistic point of view, taking into account the naive condition of our population to this novel virus strain, which reduces cross-reactive antibodies that may complicate the interpretation of the immunogenic response. Thus, our results may be relevant for any future pandemic caused by a similar virus.

A limitation of our study is the sample size which does not allow us to draw conclusions on vaccine efficacy or effectiveness based on percentage reduction of attack rates (number of new cases during the exposure period divided by the number of people in the population who could catch the disease). On the other hand, clinical attack rate was lower than that predicted by the authorities (20–40% estimated by mathematical modeling conducted in the southern hemisphere), and available data clearly indicate that the clinical protection provided by influenza vaccines is closely correlated with their immunogenicity [29]. Consequently, for influenza vaccines it is generally accepted that vaccine induced HI antibody titers, measured against influenza antigens from strains causing disease in the community, are a good surrogate marker of efficacy. In this regard, our CHC patients showed optimal response to influenza vaccine. In fact, immunologic endpoints established for seasonal influenza vaccines (proportions of seroprotection >70%, seroconversion >40% and GMTR of HI antibody titers >2.5) were largely achieved [30]. Although the size of the cohorts included in the study does not allow firm conclusions, the incidence of respiratory infections due to influenza A infection in our vaccinated population was very low.

Several factors may affect immune response including concurrent use of medications, in particular drugs influencing immune function such as immunosuppression and interferon based therapies [7], [31]. However, in our study even CHC patients under treatment with pegylated-interferon and ribavirin showed responses comparable to those seen in non-treated CHC patients and healthy controls. This is in keeping with the results obtained in a small cohort of heterogeneous hepatitis C patients [32]. In contrast, IBD patients with immunosuppression had lower immune response to pandemic (H1N1) influenza A vaccine, in agreement with other recent studies in pediatric and adult populations [8], [33], [34]. This is not surprising as data derived from seasonal influenza vaccination indicate that antibody response is diminished in immunosuppressed transplant recipients [32], [35], patients receiving chemotherapy [36] and human immunodeficiency virus infected adults [37]. We did not find differences between subjects receiving monotherapy immunosuppression and those receiving combined immunosuppression, although the study was not powered to find differences [34].

Finally, we investigated the tolerance of the influenza vaccine. Despite the fact that subjects receiving adjuvanted vaccines tended to show more adverse effects [19], [38], even in the worst-case scenario like ours, tolerance of our overall study population to the vaccine can be considered to be satisfactory, as VAPI questionnaire items scored low. Although in this study CHC patients with ongoing treatment had the highest scores concerning injection site reactions with statistically significant differences, we cannot rule out a bias due to the well known local effects that pegylated-interferon cause in nearly two-thirds of treated patients [39]. This limitation is difficult to overcome. However, the clinical relevance of these differences is likely to be minimal. In fact, considering the VAPI questionnaire questions specifically evaluating this issue, overall acceptance was satisfactory. As a further illustration of this, the willingness to be vaccinated the following year was not affected by the local reactions. This is of interest as these patients appeared to have optimal immune response to the vaccine, achieving in our limited sample size the three immunologic thresholds approved by the European Medicines Agency [30]. Vaccination was well tolerated by IBD patients, in agreement with recent data [40]. No deaths or serious vaccine-related adverse events, including neurological and autoimmune disorders in accordance with a recent published study [41], were reported during follow-up of all subjects included.

Influenza vaccination apparently did not influence the CHC therapy response. Both groups had similar prognostic factors of favorable outcome after treatment, although caution should again be exercised considering our small sample size. CHC and influenza has aroused interest because of T cell response cross reactivity of a hepatitis C virus epitope (NS3-1073) and influenza A epitope (NA-231), which may theoretically contribute to viral clearance [42]. However, this specific effect of the vaccine on CHC response was not an objective of our study and definite conclusions cannot be drawn due to our small sample size.

In conclusion, in our cohort there appeared to be no differences between CHC patients and healthy controls in serological response and acceptance of (H1N1) influenza vaccination.
